# TAL-SRX: an intelligent typing evaluation method for KASP primers based on multi-model fusion

**DOI:** 10.3389/fpls.2025.1539068

**Published:** 2025-02-18

**Authors:** Xiaojing Chen, Jingchao Fan, Shen Yan, Longyu Huang, Guomin Zhou, Jianhua Zhang

**Affiliations:** ^1^ National Agriculture Science Data Center, Agricultural Information Institute, Chinese Academy of Agricultural Sciences, Beijing, China; ^2^ National Nanfan Research Institute, Chinese Academy of Agricultural Sciences, Sanya, China; ^3^ Institute of Cotton Research of Chinese Academy of Agricultural Sciences, Anyang, China; ^4^ Hainan Yazhou Bay Seed Laboratory, Sanya, China

**Keywords:** KASP fractal evaluation, multi-model fusion, stacking integration, deep learning, hyperparameter tuning

## Abstract

Intelligent and accurate evaluation of KASP primer typing effect is crucial for large-scale screening of excellent markers in molecular marker-assisted breeding. However, the efficiency of both manual discrimination methods and existing algorithms is limited and cannot match the development speed of molecular markers. To address the above problems, we proposed a typing evaluation method for KASP primers by integrating deep learning and traditional machine learning algorithms, called TAL-SRX. First, three algorithms are used to optimize the performance of each model in the Stacking framework respectively, and five-fold cross-validation is used to enhance stability. Then, a hybrid neural network is constructed by combining ANN and LSTM to capture nonlinear relationships and extract complex features, while the Transformer algorithm is introduced to capture global dependencies in high-dimensional feature space. Finally, the two machine learning algorithms are fused through a soft voting integration strategy to output the KASP marker typing effect scores. In this paper, the performance of the model was tested using the KASP test results of 3399 groups of cotton variety resource materials, with an accuracy of 92.83% and an AUC value of 0.9905, indicating that the method has high accuracy, consistency and stability, and the overall performance is better than that of a single model. The performance of the TAL-SRX method is the best when compared with the different integrated combinations of methods. In summary, the TAL-SRX model has good evaluation performance and is very suitable for providing technical support for molecular marker-assisted breeding and other work.

## 1 Introduction

The kompetitive allele-specific PCR (KASP) technique is capable of realizing the precise identification of site-specific SNP (single nucleotide polymorphism) double allele genotypes in different species genome sample types (Wang et al., 2020), and is widely used in molecular marker-assisted selective breeding, quality testing, variety identification, and stress assessment, etc., because of its unique advantages of flexibility, high efficiency, and low cost (Tang et al., 2022). However, population genotype amplification and segregation are complex and variable, and the evaluation of typing results directly affects the efficiency of KASP marker development (Zhi et al., 2024). Therefore, it is necessary to realize the intelligent and accurate evaluation of the relative independence of population genotyping results, in order to scale up the screening of excellent KASP markers and to improve the efficiency of marker development.

Up to now, there are three main methods for evaluating the typing results in studies utilizing competitive allele-specific PCR technology. The most widely used method is the manual visual judgment of genotyping, which is mainly observed and recognized by agricultural experts or technicians. Due to the flexible and changeable performance of the typing results, the application of this method in breeding practice requires that professionals must have long-term and rich experience in reading typing diagrams, and spend a great deal of time in order to select well-typed KASP markers, so the evaluation process is accompanied by the problems of time-consuming, subjective, and large-scale material validation. For example, Yu et al. (2023) directly observed the fluorescence typing status of different colored dots in the KASP fluorescence detector in the screening and validation of candidate core markers for genotype identification of tobacco varieties, and used the subjectively evaluated well-typed SNP sites as molecular markers in their subsequent studies; Schoonmaker et al. (2023) in the validation of cotton leafroll virus resistance gene association markers, combined with the observation results, proposed that the pure and heterozygous group separation, the cluster within the tight pattern can be proved that the markers are good; Zhao et al. (2023) in the development of Pi2 KASP marker for rice blast resistance gene, because the visual typing results were not clear enough, they further set up negative and positive controls, and initially judged that the marker was feasible after observation. However, because the typing results were scattered, four additional cycles were added to the original testing program to obtain more intuitive and clearer KASP genotyping results; Kalendar et al. (2022) used grid lines with parallel horizontal and vertical axes to partition the KASP genotyping map in an experiment to identify alleles of barley varieties with known genotypes, and observed whether pure and heterozygous genotypes were located in different partitioned regions within the map, respectively, in order to evaluate the accurate validity of the markers. The second category is the method of quantitative assessment of indicator values, although with the development of new SNP genotyping techniques, some scholars have proposed the use of ANOVA to quantitatively assess the differences in indicator values in comparison tests between KASP and TaqMan and other techniques. However, the criteria proposed by this method to use the relative height of the index value to judge the good or bad typing effect are vague, and the application scope in KASP test is limited (Broccanello et al., 2018). The third category is the traditional machine learning approach, usually the SNP genotyping results data sample size is large, the data dimension is high and has the complexity of non-linear relationships, compared with the statistical analysis using a small number of indicators, machine learning as a powerful data-driven framework is more suitable for providing accurate solutions to the complex relationships between a large number of variables in the KASP test results (Kok et al., 2021), such as Chen et al. (2024) proposed an intelligent typing evaluation model for KASP marker primers, which is based on the typing effect level evaluation criteria, introduces K-Means clustering algorithm in the design module to fit the gene population aggregation and classification effects, and finally realizes the intelligent typing result evaluation by logical decision tree algorithm.

However, there are relatively few relevant studies on intelligent typing evaluation of KASP, and the existing evaluation criteria for typing effect levels lack more detailed classification hierarchies, which may lead to a low identification rate of good markers for large-scale screening, thus resulting in a large amount of wasted financial and material resources, which collectively limit the application of KASP technology in assisted breeding work. Second, shallow learning models still have bottlenecks in handling big data, and given that deep learning, as one of the most popular data-driven methods, it may be a useful exploration to apply it to automatically extract and learn the intrinsic features of KASP trial result data (Du et al., 2019; Ahmed et al., 2023).

Based on the above considerations, we propose TAL-SRX, an intelligent typing evaluation method for KASP priming based on multi-model fusion. We utilize the Stacking integrated learning framework to synthesize and apply multiple heterogeneous base learners, and construct a two-layer structure to combine and train with the eXtreme Gradient Boosting (XGBoost) model to drive the data while improving the prediction accuracy of the model. In addition, two deep learning models are selected to be given weights and then introduced into the integrated learning framework to further enhance the ability of the model to master multidimensional features under complex task conditions. The performance of the model is tested by the results of KASP marker typing test of 3399 sets of cotton variety resource materials to verify the effectiveness of the proposed method. Our results not only provide new insights for evaluating the distribution patterns of KASP marker amplification products, but also lay an important foundation for accelerating breeding efforts to precisely localize and select target traits at the molecular level in a variety of crops.

## 2 Materials and methods

### 2.1 Experimental data

#### 2.1.1 Raw data

**Figure 1:**
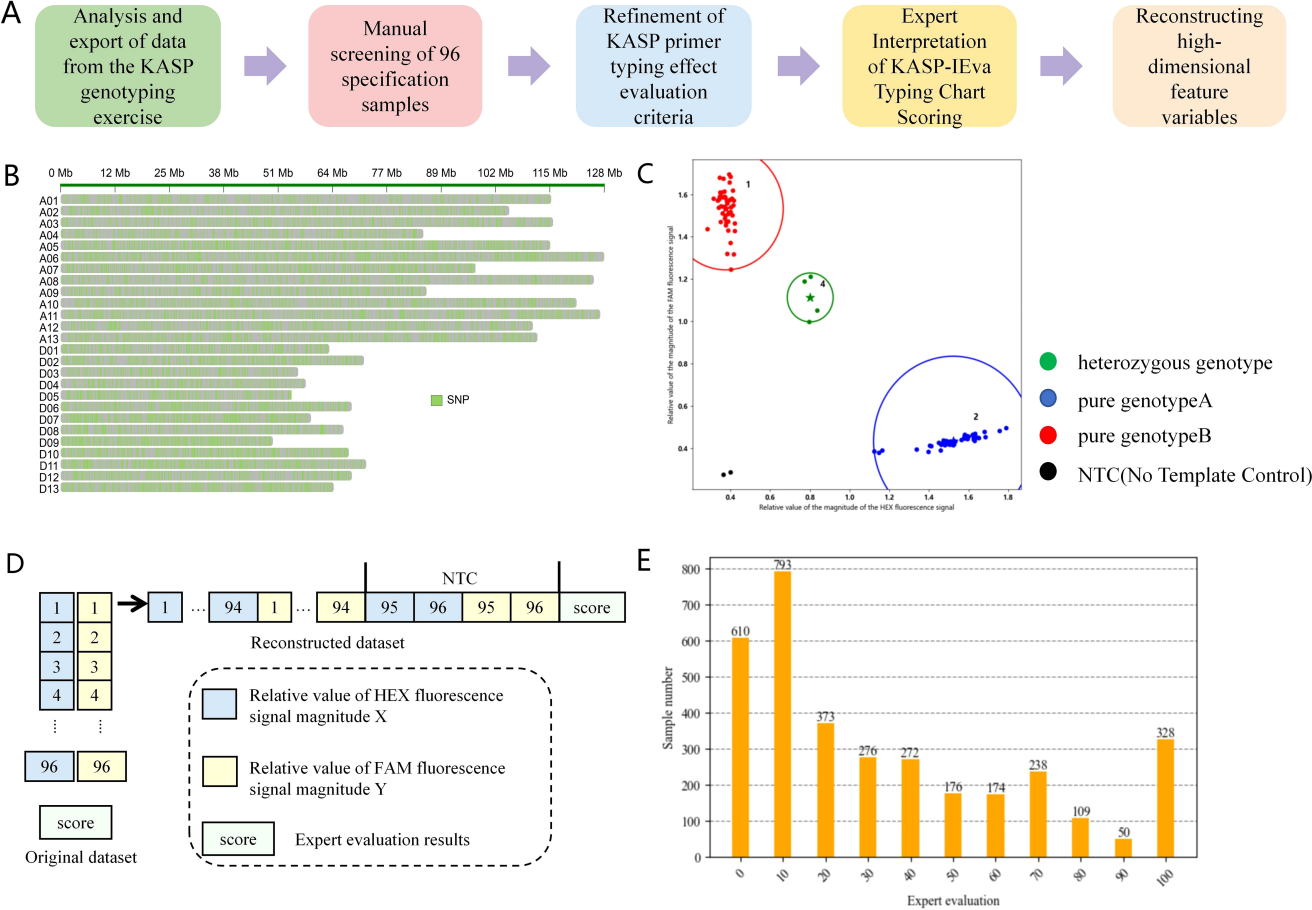
Flowchart of dataset construction. **(A)** Steps of dataset integration construction. **(B)** Schematic diagram of genome-wide distribution of SNP variants. **(C)** Example of KASP-IEva model typing diagram. **(D)** KASP marker typing report statistical data structure and sample structure of high-dimensional dataset. **(E)** Distribution of the amount of data in each score of the dataset after scored by the experts.

In this study, we selected the KASP marker typing report statistics of the resource materials of cotton varieties produced by SNPline, a high-throughput genotyping detection platform of LGC (**Figures 1A, B**), and the resource materials included resource varieties, line materials, validated varieties and genetically segregated population materials. The source of the genotyping report statistics is the Cotton Quality Supervision, Inspection and Testing Center of the Ministry of Agriculture and Rural Affairs, Cotton Research Institute, Chinese Academy of Agricultural Sciences, which contains 319 test results from different SNPs and different DNA samples from 2019 to 2023. Due to the different sample arrangements on the motherboards of each test, we extracted statistics in the format of 94 DNA samples and 2 NTC (negative control reaction without adding DNA samples in the PCR assay) assay data set to build the original dataset, and obtained a total of 3399 sets of statistics.

#### 2.1.2 Criteria for evaluating the typing effect of KASP primers

Based on a large amount of experimental data, we made a detailed division of the evaluation criteria of KASP primer typing effect, as shown in **Table 1**, and we set a scoring range from 100 to 0 to indicate the primer combination morphology from the best case to the worst case. Specifically, when the competitive primer combination morphology exhibits independent aggregation of pure and heterozygous genotypes and the pure genotypes are located at the maximum of the two axes respectively, and the heterozygous genotypes are located in the center of the line connecting the two pure genotypes, the score is 100. And the score gradually decreases as the degree of the independent aggregation of pure and heterozygous genotypes exhibited by the competitive primer combination morphology decreases, and the segregation of the genotypes decreases, and the score gradually decreases Until complete relative dispersion and inability to accurately typify, the score is 0. This criterion provides a quantitative method for evaluating the combinatorial morphology of competitive primers, and provides a powerful tool for analyzing the amplification efficiency, specificity, and combinatorial competitiveness of genotypes.

**Table 1:** Criteria for evaluating the typing effect of KASP primers.

Score	Primer combination morphology
100	NTC has no obvious specificity, pure and heterozygous genotypes are clustered independently, pure genotypes are located at the maximum of each of the two axes, and heterozygous genotypes are located in the center of the line connecting the two pure genotypes
90	NTC has no obvious specificity, pure and heterozygous genotypes are clustered independently, pure genotypes are located at the maximum of each of the two axes, and heterozygous genotypes deviate from the line connecting the two pure genotypes
80	NTC has no obvious specificity, pure and heterozygous genotypes are clustered independently, pure genotypes are located at the maximum of each of the two axes, and heterozygous genotypes are shifted along the line connecting the two pure genotypes
70	NTC has no obvious specificity, pure and heterozygous genotypes are independent, one pure genotype is shifted or trailing along the coordinate axis, and the heterozygous genotype is located in the center of the line connecting the two pure genotypes
60	NTC has no significant specificity, pure and heterozygous genotypes are independent, pure genotypes are shifted along the coordinate axis, and heterozygous genotypes are located in the center of the line connecting two pure genotypes
50	NTC has no obvious specificity, pure and heterozygous genotypes are independent, pure genotypes have a trailing tail, and heterozygous genotypes are located in the center of the line connecting the two pure genotypes
40	NTC is not clearly specific, pure and heterozygous genotypes are partially diffuse or shifted along the axes, but can be typed
30	NTC is not clearly specific, all pure genotypes are relatively diffuse and heterozygous genotypes are shifted, but can be barely differentiated
20	NTC has no obvious specificity, all pure and heterozygous genotypes are relatively diffuse, some genotypic loci are crossed and cannot be accurately typed
10	NTC shows marked specificity
0	Pure and heterozygous genotypes are all relatively diffuse, genotypic loci are crossed and cannot be typed

#### 2.1.3 Data set construction

The SNP site number, HEX fluorescence signal magnitude relative value X, FAM fluorescence signal magnitude relative value Y and the sample number in the original data set were taken to apply the KASP-IEva model for typing (**Figure 1C**), and the results of the expert’s scoring of the typing effect were used as the criteria. Finally, using the SNP locus number as the number of each data set, the HEX fluorescence signal magnitude relative value X and FAM fluorescence signal magnitude relative value Y of each group were combined and reconstructed into 192 high-dimensional feature variables, and the expert scores were used as the labeling categories, which together comprise the model dataset (**Figures 1D, E**), of which 84% was used as the training set and 16% as the test set to evaluate the model validity. **Table 2** is the sample of the model data set. Fusion method in the Stacking model will be cross-validated using five folds of the training set from which the validation subset will be further divided.

**Table 2:** Model data set samples based on KASP marker typing results.

SNP site number SNPID	Relativevalue of HEX fluorescence signal magnitude X and relativevalue of FAM fluorescence signal magnitude Y							Label
	1	2	3	-	190	191	192	
CS01	0.34152	1.23918	0.31628	-	0.36266	0.29725	0.31885	50
CS02	1.23677	1.20181	0.32964	-	0.35707	0.29542	0.29948	30
CS03	0.35668	1.18205	1.20185	-	0.51436	0.96719	0.86575	10
CS04	0.36522	0.31756	1.09486	-	0.36946	0.28544	0.29809	40
CS05	1.42639	0.32153	1.42668	-	0.3707	0.29418	0.28624	60
CS06	1.40921	0.46171	1.41027	-	0.3593	0.30589	0.32609	100
CS07	0.36557	0.32076	0.32602	-	0.39087	0.30391	0.31229	30
CS08	0.71165	0.32425	0.31644	-	0.35485	0.2853	0.2939	50
CS09	1.20593	0.31494	0.31246	-	0.34687	0.28328	0.29112	100
CS10	0.41205	0.38226	0.37761	-	0.35003	0.28565	0.30938	40

### 2.2 TAL-SRX multi-model fusion evaluation methodology

#### 2.2.1 TAL-SRX architecture

**Figure 2:**
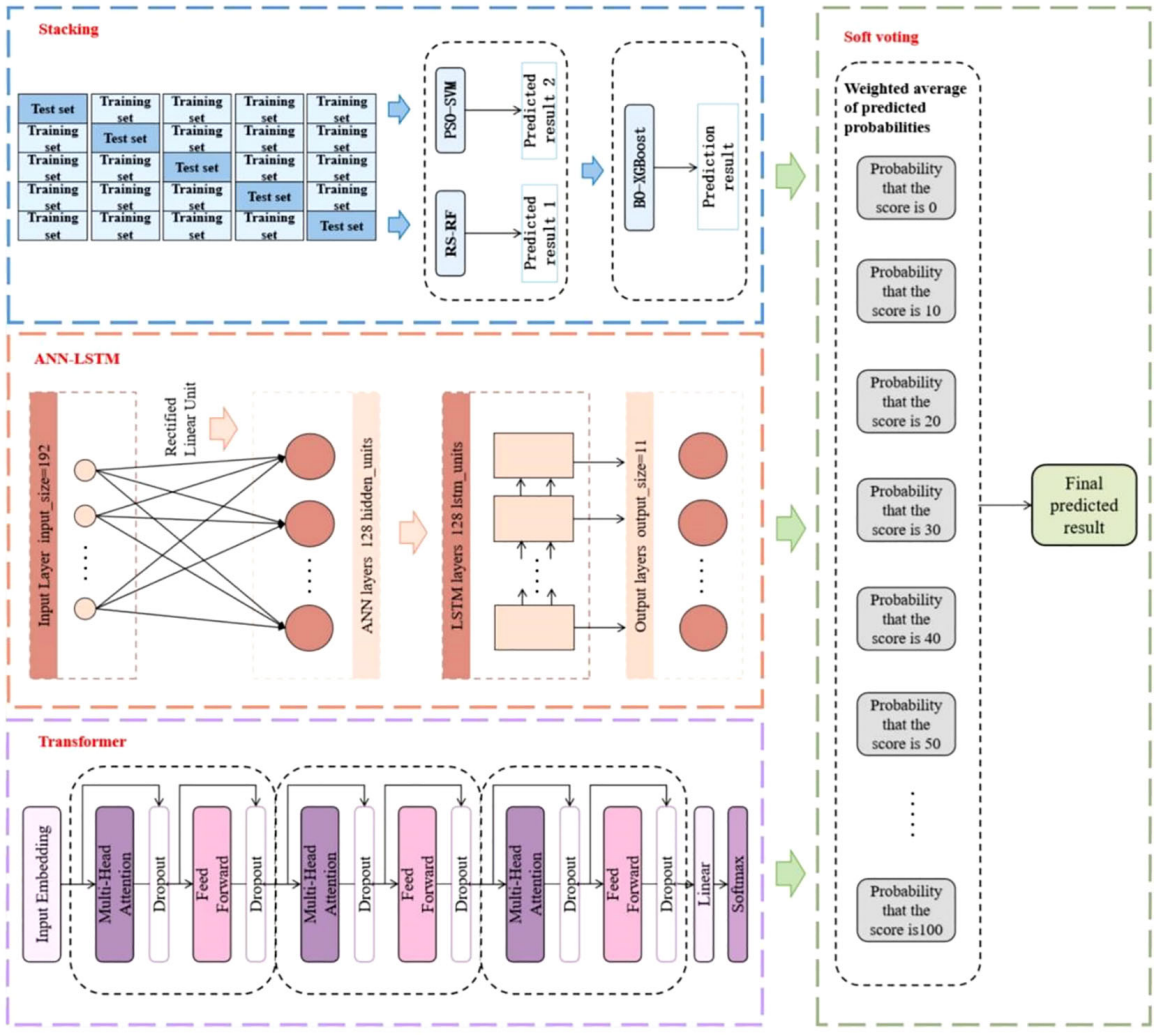
Overall architecture of TAL-SRX multi-model fusion approach.

In order to improve the accuracy of good markers screening recognition at scale, this paper selects six machine learning algorithms, namely Support Vector Machine (SVM), Random Forest (RF), eXtreme Gradient Boosting (XGBoost), Artificial Neural Network (ANN), Long Short-Term Memory (LSTM) and Transformer. Two integrated strategies of Stacking and Soft Voting are used for algorithm combination to construct a multi-model hybrid learning method driven by statistical data for KASP marker typing report, which overcomes the defects of a single learning model, enhances the functional nature of feature parsing of high-dimensional data, and possesses a powerful, stable, and comprehensive learning capability. **Figure 2** illustrates the flow of the KASP primer typing effect evaluation method.

The TAL-SRX multi-model fusion evaluation method makes full use of the advantages of traditional machine learning algorithms and deep learning algorithms. Firstly, the “base model-metamodel” approach is used to connect the three traditional machine learning algorithms. In order to accelerate the parameter search process and maximize the performance of each learner, different optimization algorithms are used to meet the unique tuning requirements of different learners. In the Stacking integrated learning framework based on hyperparameter optimization algorithms, the heterogeneous learner parses features of different dimensions within the dataset to generate new feature variables of lesser dimensions, and the meta-learner integrates the data and thus predicts the probabilities. Then the deep learning algorithm is introduced, and the data output processed by ANN is passed to the LSTM layer to flexibly capture complex features while enhancing the model’s ability to adapt to heterogeneous samples, and then the Transformer model based on the self-attention mechanism is added to capture the global dependencies between the input variables and enhance the model robustness. Finally, the voting integration model extracts the shared features of the output data of each basic algorithm and obtains the evaluation results of KASP primer typing effect.

#### 2.2.2 Stacking integration based on hyperparameter optimization algorithm

**Figure 3:**
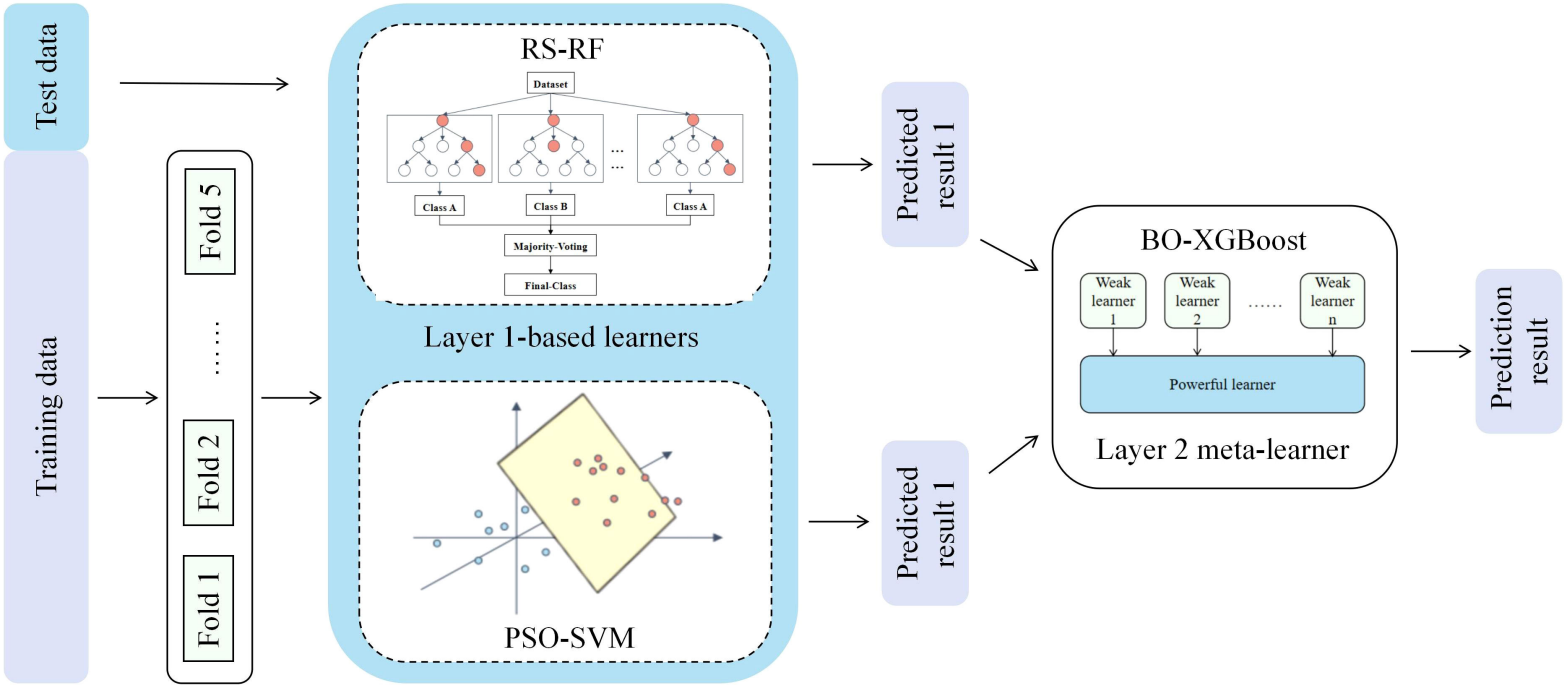
Stacking integrated learning evaluation model.

Stacked integration is a widely used integration learning technique, the basic principle of which is to make predictions through a two-layer nested structure (He et al., 2024; Shi et al., 2023). In this paper, the stacked model trains two base learners and uses their prediction results as inputs to the meta-learner to fully exploit the original dataset features to improve the prediction accuracy. For each sub-model, an optimization algorithm is used to improve the model structure, and RS-RF, PSO-SVM and BO-XGBoost are constructed respectively to find the hyper-parameter combinations with the best performance, **Figure 3** illustrates the overall framework of Stacking, and the specific implementation process is (Fu et al., 2020):

(1) Data preprocessing is performed on the training set S = {(y_n_, x_n_), n = 1,…, N} by using the five-fold cross-validation method, which randomly divides the data set S into five equal-sized and disjoint subsets S1, S2,…, S5; (2) one subset is selected as the validation set and the remaining four subsets are used as the training set, and the PSO-SVM model is trained on the training set, and the prediction is performed on the validation set and the prediction result is saved. And then select the remaining four subsets sequentially as validation sets, respectively, and finally obtain five prediction results, which are combined into the set Z1; (3) For the RS-RF model, repeat the operation of step (2) to obtain the set Z2; (4) Combine the prediction results of the base learner in the five-fold cross-validation, and horizontally splice them into the new feature variables Z = {Z1, Z2}, thus realizing the feature conversion from the base learner to the meta-model, and train the meta-model by using these new features and the original labels y_n_; (5) After the model training is completed, use the base model to predict the test set, and the prediction results are input to the meta-model to obtain the final KASP primer typing effect evaluation results (Liu et al., 2024). The base and meta learners are described in detail in the subsequent subsections.

##### 2.2.2.1 RF model based on RS optimization

Random Search (RS) finds the best model parameters by randomly sampling multiple parameter combinations in a predefined parameter space and evaluating the performance of each combination (Shakya et al., 2024). It eliminates the need for gradient information and can explore globally to avoid local optimization.

RF is an algorithm that integrates multiple decision trees based on the idea of bagging (Breiman, 2001). In RF, many trees are constructed using a randomly selected training dataset and a random subset of predictor variables, and the results of each tree are aggregated using the absolute majority voting method to obtain the prediction categories (Speiser et al., 2019). In this paper, we optimize the modeling process of RF based on RS by extracting a sample subset through Bootstrap method, randomly selecting a subset from the sample features to find the optimal splitting point when each node splits, and constructing multiple unpruned decision trees using these sample and feature subsets, and evaluating the performance of the model in each iteration to select the optimal hyper-parameter configurations. Where the absolute majority voting strategy is formulated as:


(1) $$H\left(x\right)=\left\{ \begin{array}{l} \;c_{j},\;\;\;\;if\sum_{i=1}^{T}h_{i}^{j}\left(x\right)>0.5\sum_{k=1}^{N}\sum_{i=1}^{T}h_{i}^{k}\left(x\right);\\ reject,\;\;\;\;otherwise. \end{array}\right.$$

Where *h_i_* is the base learner, i.e., the decision tree, *c_j_* is the category labeling, *T* is the total number of base learners, $h_{i}^{j}\left(x\right)$ is the output of *h_i_* on the category labeling *c_j_*, and N is the $h_{i}^{j}\left(x\right)$ total number of predicted outputs of *h_i_* on sample x.

Each tree in RF has different segmentation features and segmentation points, which has stronger nonlinear data processing ability and overfitting resistance compared with a single decision tree model, and meanwhile, stochastic search can help RF model better adapt to the dataset and improve its robustness and generalization ability.

##### 2.2.2.2 SVM model based on PSO optimization

Particle Swarm Optimization (PSO) originates from the phenomenon of bird flock foraging (Kennedy and Eberhart, 1995), and its essence is to simulate the intelligent behavior of the group, through the information sharing and mutual learning between individuals and groups, each particle adjusts its own position and speed in the search space (Tang et al., 2023), and gradually finds the optimal solution of the problem.

The core idea of SVM is to classify data by solving the maximum margin hyperplane in the feature space, a method known as maximum interval classification. When confronted with linearly indivisible data, SVM maps the data to a higher dimensional space by a kernel method so that it becomes linearly divisible in this new space (Utkin et al., 2016). In this paper, we use the PSO algorithm to optimize the parameters of the SVM modeling process, and determine the optimal parameter model by evaluating the fitness of each particle and updating the optimal positions of the individuals and populations in each iteration, where the decision function of the SVM can be expressed as:


(2) f(x)=w·ϕ(x)+b

Where *x* is the input eigenvector, *ϕ(x)* is the feature map (defined by the kernel function) mapping the input eigenvector *x* to the high-dimensional space, w is the normal vector (weight vector) of the hyperplane found in the eigenspace, *b* is the bias term, and the inner product *w·ϕ(x)* denotes the projection into the eigenspace.

SVM has excellent adaptability to class imbalance problems with large feature differences, and the PSO algorithm, with its outstanding performance of simple implementation, high accuracy and fast convergence, can help SVM to improve its ability to handle high-dimensional complex datasets (Luo et al., 2023).

##### 2.2.2.3 XGBoost model based on BO optimization

Bayesian Optimization (BO) emerges at the forefront of black-box optimization methods due to its high efficiency in finding the global optimal solution with fewer times, and its advantage lies in its ability to infer the posterior distribution of the objective function based on the *a priori* information and the results that have already been observed, achieving a good balance between exploration and exploitation in the search process 0 (Wang et al., 2023).

XGBoost is a scalable end-to-end tree boosting technique in Boosting integrated learning model (Dong et al., 2022; Chen and Guestrin, 2016). Its objective function consists of two parts: the loss function and the regular term, and the core idea of the model is to measure the deviation through the loss function, and use the regular term to control the complexity of the model to avoid overfitting while reducing the deviation. In this paper, we use BO to optimize the modeling process of XGBoost by evaluating the performance of sampling points and updating the Gaussian process agent model in each iteration to select the optimal parameter combinations, so as to gradually approximate the optimal solution of the unknown objective function, where the objective function is defined as follows (Talukder et al., 2024; Cai et al., 2021):


(3) $$L\left(\phi \right)=\sum_{i=1}^{n}l\left(y_{i},{\hat{y}}_{i}\right)+\sum_{k=1}^{K}\text{Ω}\left(f_{k}\right)$$

Where *l* is the loss function, *Ω* is the regularity term, $y_{i}$ is the true value, ${\hat{y}}_{i}$ is the predicted value, and $f_{k}$ denotes each tree.

XGBoost integrates multiple weak learners into a single strong learner with higher computational speed and better model performance, and BO optimization improves the stability of the XGBoost model on the dataset and reduces the risk of overfitting or underfitting.

#### 2.2.3 Voting integration incorporating deep learning algorithms

Since the KASP marker typing report statistics are produced from different batches, in order to avoid the threat of genetic data heterogeneity to the model robustness, this paper proposes an integration method that introduces the deep learning models ANN-LSTM and Transformer on top of the integration of traditional machine learning algorithms, which will be described in detail in the following two deep learning frameworks:

1. ANN-LSTM: ANN is a class of computational models inspired by biological neural networks, which are widely used in tasks such as data classification (Yang and Wang, 2020; Affonso et al., 2015). Combined with LSTM, we construct a hybrid neural network that uses ANN as a feed-forward neural network layer, integrating the nonlinear relationship capturing ability of ANN and the complex data modeling ability of LSTM (Greff et al., 2016).In the forward propagation process, the input data first passes through the ANN layer, which uses ReLU as the activation function for nonlinear transformation to increase the expressive ability of the model, and the data passes through the 128 hidden units of the layer to adjust the shape of the output after the initial feature extraction, and the LSTM layer is also set up with 128 units, which is able to efficiently capture the intrinsic complex structure of the data by means of the mechanism of the memory unit and the forgetting gate. Finally, the output of LSTM is mapped to the target category space. In the backpropagation process, the cross-entropy loss function is calculated, and the model parameters are updated in training rounds (epochs) by the Adam optimizer. After training, the model is predicted on the test set and the probability distribution of each score is calculated.
2. Transformer: the Transformer is a neural network based mostly on self-attentive mechanisms for capturing global dependencies between input features and focusing on key details of the data (Han et al., 2021). In this architecture, an encoder with feature extraction capability is first defined and the input data is processed through three encoder layers to increase the model depth. During forward propagation, a self-attention mechanism and a feed-forward neural network process the data to capture higher level relationships and extract more complex features. To prevent the model from overfitting, a Dropout layer is added to randomly discard some neurons, and finally the processed features are mapped to the target category space by a linear layer. During the training process, the model calculates the cross-entropy loss by backpropagation and updates the model parameters using the Adam optimizer, and during the testing phase, the model calculates the score category probability for each sample by softmax function and outputs it.

The prediction probabilities of the three models, ANN-LSTM, Transformer and Stacking, are weighted and averaged to obtain the results of the KASP primer typing effect evaluation. With this approach, we obtain a powerful and stable system that contains multiple heterogeneous base learners, and TAL-SRX has the ability to adapt to different scenarios compared to a single prediction model, thus obtaining better prediction results (Ribeiro and dos Santos Coelho, 2020).

## 3 Test results and analysis

### 3.1 Test environment

The operating system environment was Windows 11 with a 12th Gen Intel(R) Core(TM) i5-12500 3.00 GHz CPU, 32.0 GB of RAM on board, and a GPU of NVDIA GeForce RTX 3080, 10 GB of RAM. The training environment was created by Anaconda3 and the environment was configured with Python 3.10.13 and PyTorch2.0.0 deep learning framework.

### 3.2 Indicators for model assessment

In order to verify the effectiveness of the proposed modeling method, the model performance is evaluated using accuracy, precision, recall, F1 score and Cohen’s Kappa coefficient. The value range of the first four indicators is 0-1, and each scoring category is calculated separately, and then the macro-averaging (Macro-averaging) method is used to obtain the average indicator values, and the evaluation formulas for each category are as follows:


(4) $$Accuracy\;=\;\frac{TP}{TP+FP+FN}$$

(5) $$Precision=\frac{TP}{TP+FP}$$

(6) $$Recall\;=\frac{TP}{TP\;+\;FN}$$

(7) $$F1score=\frac{2\;\times \;\left(Precision\;\times \;Recall\right)}{Precision\;+\;Recall}$$

Where *TP*, *FP* and *FN* denote i.e., the number of samples correctly predicted to be that label, the number of samples incorrectly predicted to be that label and the number of samples incorrectly predicted to be other labels, respectively.

The Kappa coefficient provides a more reliable measure of consistency than simple accuracy by taking into account the contingency factor of classification, with values ranging from -1 to 1 on the following scale: 0.81 - 1.00 indicates almost perfect agreement; 0.61 - 0.80 indicates significant agreement; 0.41 - 0.60 indicates moderate agreement; 0.21 - 0.40 indicates fair agreement; 0.00 - 0.20 indicates very low agreement; less than 0 indicates no agreement or very poor agreement. The formula for Kappa coefficient is given below:


(8) $$k=\frac{p_{o}-p_{e}}{1-p_{e}}$$

Where *p_o_* denotes the proportion of predicted labels that are consistent with actual labels, and *pe* denotes the proportion of consistency under the assumption that predicted and actual labels are stochastically independent.

### 3.3 Model hyperparameter selection and model performance analysis

The selection of the hyperparameters of the base model is crucial for the improvement of the prediction performance of the integrated model (Yang and Shami, 2020), in order to ensure that the performance of TAL-SRX tends to be optimal, RS is used to optimize the RF model, PSO is used to optimize the SVM model, BO is used to optimize the XGBoost model, the hyperparameter optimization of ANN-LSTM and Transformer are both using trial and error method, and the determination of the voting weights is using the grid search method, and the optimization of the hyperparameters is performed by the grid search method. The parameter optimization of each model is shown in **Table 3**.

**Table 3:** Core hyperparameters of the base model.

Model name	Hyperparametric configuration
RF	n_estimators=202, max_depth=34, min_samples_split=5, min_samples_leaf=1
SVM	C=695.65, kernel=“rbf”, Gamma=0.0089
XGBoost	colsample_bytree=0.87, learning_rate=0.29, max_depth=7, n_estimators=139, subsample=0.83
ANN-LSTM	lr=0.01, epochs=500
Transformer	nhead=6, dropout=0.01, lr=0.001, epochs=500
TAL-SRX Soft Voting	weight_transformer=0.25, weight_stacking=0.95, weight_ANN-LSTM=0.55

**Figure 4:**
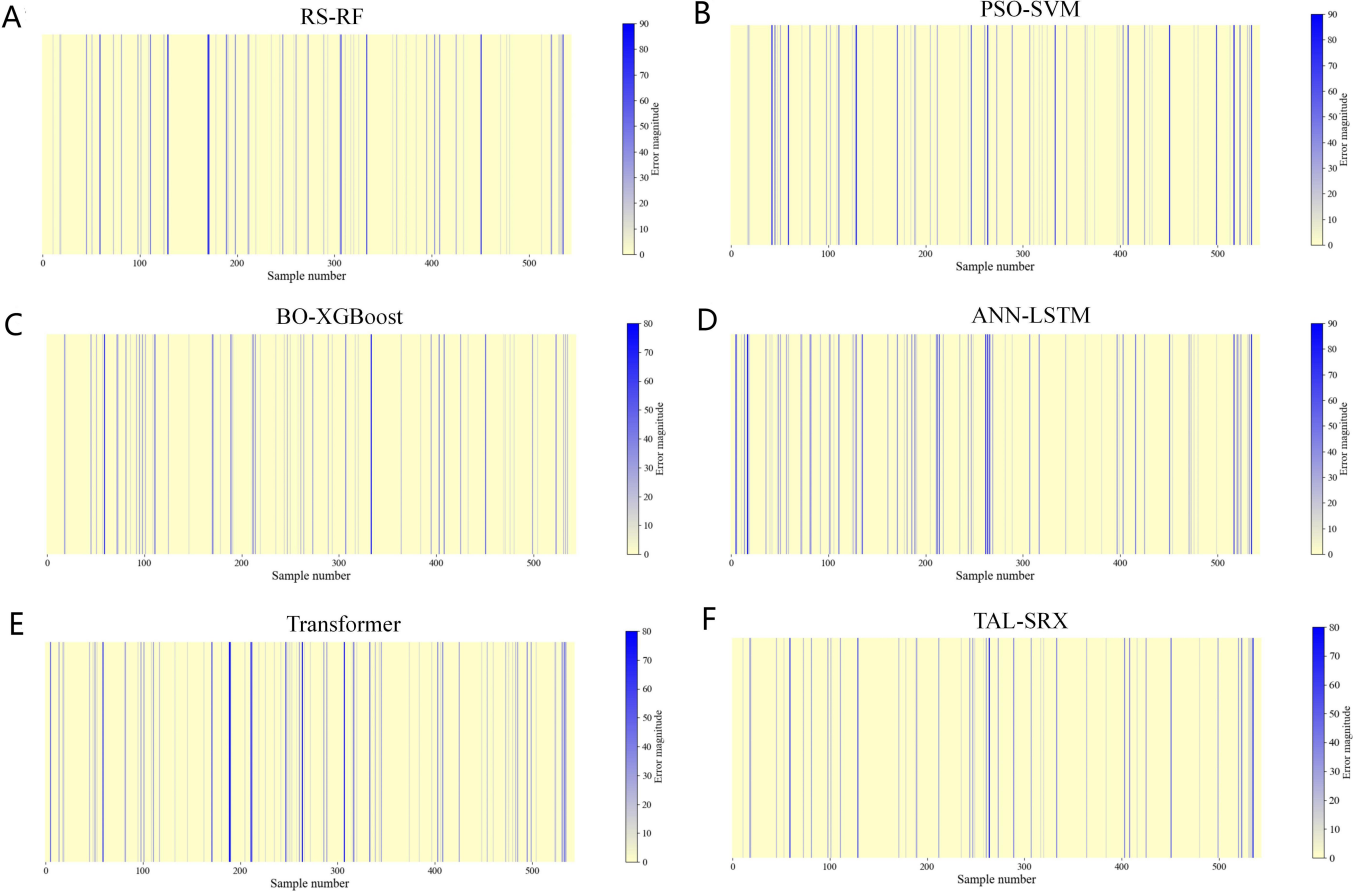
Error heatmap of the base model and the TAL-SRX approach. **(A)** Error heatmap of RS-RF. **(B)** Error heatmap of PSO-SVM. **(C)** Error heatmap of BO-XGBoost. **(D)** Error heatmap of ANN-LSTM. **(E)** Error heatmap of Transformer. **(F)** Error heatmap of TAL-SRX.

The prediction performance of the base model is improved to some extent after optimization, and the TAL-SRX integrated strategy obtains a more comprehensive learning prediction result for the dataset by integrating diverse base algorithms and observing the data space and structure from different perspectives. Comparative analysis of test set prediction results between the base model and the TAL-SRX integrated algorithm, **Figure 4** shows the error size between the predicted value and the actual value of each model, which intuitively demonstrates which samples have been incorrectly evaluated and classified in the prediction process of the model, and the color bar on the right side of the heatmap indicates the correspondence between the color and the size of the error, with the topmost displaying the color of the largest error, and the bottommost being the color of the smallest error. As can be seen from the figure, the TAL-SRX algorithm has fewer blue bars and generally lighter colors, which indicates that the TAL-SRX algorithm has a lower evaluation error rate than the other base models, and the error values are generally smaller than those of a single model.

**Table 4** compares the prediction performance of the base model with the TAL-SRX algorithm, and the accuracy of the five single models ranges from 84.93% to 89.15%, which verifies the feasibility of the five algorithms, including RS-RF, SO-SVM, and BO-XGBoost, as the base learner. The accuracy of our proposed TAL-SRX integration algorithm is 92.83%, which is 3.68% higher than the PSO-SVM model with the highest accuracy among the single models, and the precision, recall, and F1 score of this method are 93.82%, 87.65%, and 89.88%, respectively, which are higher than that of the five single models, indicating that the TAL-SRX integration method has a better performance than the base model The integrated performance is significantly improved and has high prediction accuracy.

**Table 4:** Comparison of base model and TAL-SRX test set performance.

Model	Accuracy(%)	Precision(%)	Recall(%)	F1 Score(%)
RS-RF	88.97	93.2	84.51	87.71
PSO-SVM	89.15	92.8	84.34	87.41
BO-XGBoost	88.97	90.91	83.89	86.38
ANN-LSTM	84.93	86.09	82.2	83.01
Transformer	86.58	84.95	82.71	83.48
TAL-SRX	92.83	93.82	87.65	89.88

**Figure 5:**
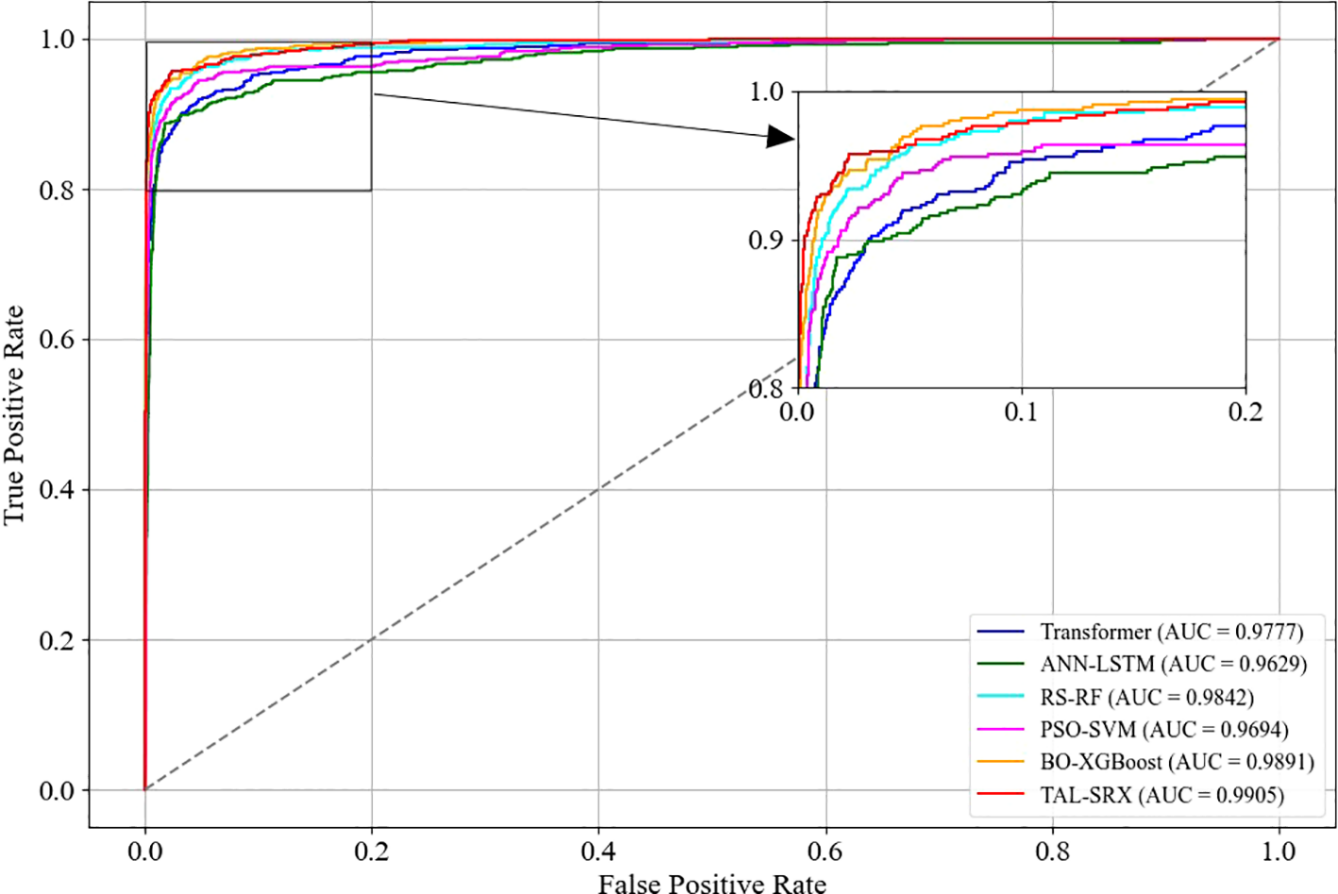
ROC curves for the base model and the TAL-SRX approach.

In order to reflect the model’s ability to distinguish between different labels, the ROC curve is drawn to calculate the average AUC value of each model in the case of multiple categories. As shown in **Figure 5**, the dashed line is the baseline, and the AUC represents the area below the ROC curve. The farther the ROC curve is from the baseline, the larger the AUC is, indicating that the model has a stronger ability to distinguish between samples. Among the six evaluation algorithms, the base model BO-XGBoost has the second highest AUC value of 0.9891, and the TAL-SRX strategy has the highest AUC value, which is 0.0014-0.0276 higher than that of the single learner, and on the whole, the TAL-SRX can effectively recognize and evaluate the KASP typing samples with different labels.

**Figure 6:**
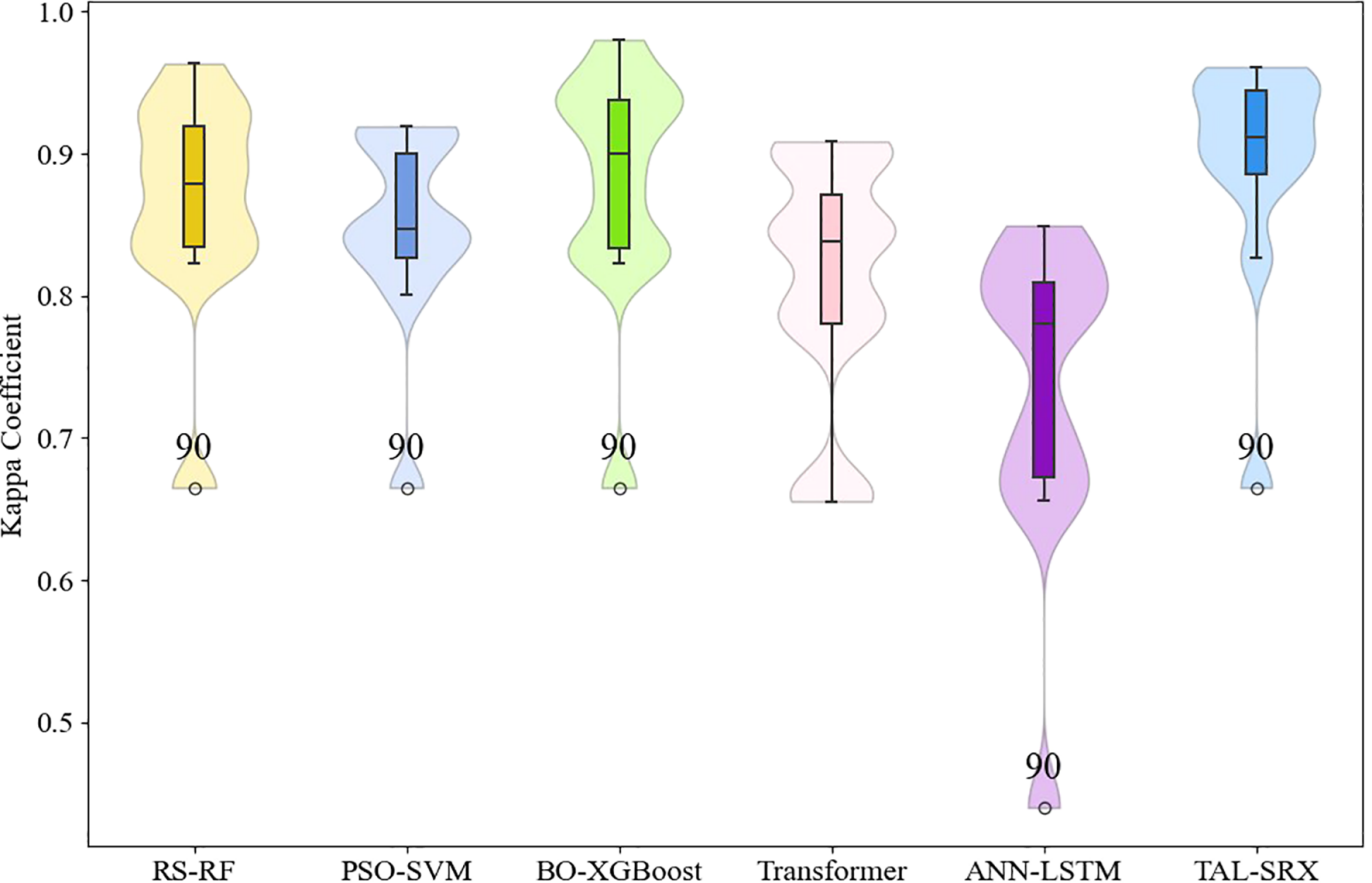
Kappa coefficients for the base model and the TAL-SRX approach.

The models are further analyzed for different label classification consistency, and the box violin plot of Kappa coefficient for each model is shown in **Figure 6**. It can be seen that the median Kappa coefficient of each single model is distributed between 0.7 and 0.9, and the distribution is denser near 0.8. After applying the integrated method, the median Kappa coefficient of TAL-SRX is higher than 0.9 and densely distributed near 0.9, and the overall distribution pattern is tighter, which is a very significant improvement, indicating that the predicted labels of the TAL-SRX method are more consistent with the actual labels are more consistent and the model is more stable. Among them, the anomalous value of Kappa coefficient for 90-point labels is affected by the uneven distribution of samples in the dataset.

In summary, the experimental results prove that compared with the base model, the TAL-SRX method has higher evaluation accuracy, consistency and stability. Analyzing from the theoretical point of view, on the one hand, TAL-SRX makes full use of the differences of the heterogeneous models and takes advantage of the powerful nonlinear modeling ability of the base model, so as to be able to comprehensively capture the detailed characteristics of the data, and, on the other hand, the base model takes advantage of the differences. For example, BO-XGBoost is based on the gradient boosting framework, which enhances its generalization ability and robustness to noisy data by controlling the depth of the tree, and the characteristics of each internal model enhance the integrated expression ability of TAL-SRX, so that TAL-SRX has obvious advantages over a single model, and is able to achieve effective prediction of the typing effect of KASP primers.

### 3.4 Comparative analysis of integrated combined approach ablation

In order to demonstrate the effectiveness of the two integration strategies in TAL-SRX, ablation comparison tests are performed using different integration combination approaches. Firstly, Stacking integration and deep learning model integration using traditional machine learning algorithms alone are used for prediction, in which the integration combinations of ANN-LSTM and Transformer are used to determine the best weight combinations using grid search, which are 0.45 and 0.4, respectively, and, secondly, the ANN-LSTM integration is added on top of Stacking, and the best weight combinations are determined using grid search weight combinations were 0.25 and 0.05, while the Transformer integration was added on top of Stacking and grid search was used to determine the optimal weight combinations of 0.35 and 0.1.

**Figure 7:**
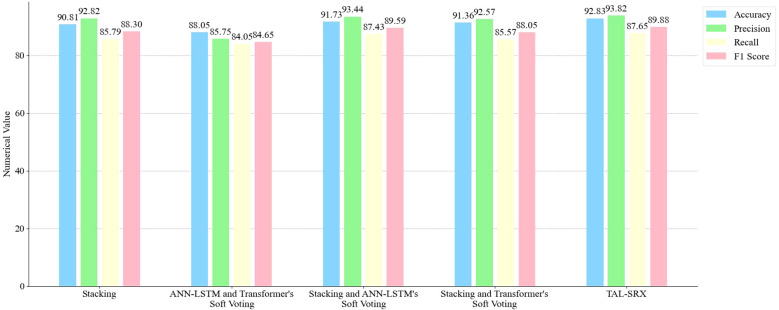
Performance comparison of different integration combinations.

The test of each integration combination method is shown in **Figure 7**, which shows that compared to the Stacking integration using traditional machine learning algorithms alone, the accuracy, precision, recall and F1 score of TAL-SRX are 2.02%, 1.00%, 1.86% and 1.58% higher, respectively, which indicates that the deep learning algorithms have the ability to extract high-level features and key details when processing complex data is stronger. Compared to the integrated combination of deep learning alone, the accuracy, precision, recall, and F1 score of TAL-SRX are 4.78%, 8.07%, 3.60%, and 5.23% higher, respectively, which is attributed to the fact that Stacking integrates the advantages of more learners and reduces the bias due to data segmentation through five-fold cross-validation in the model training stage, thus improving the prediction Accuracy. In addition, compared to the simple Stacking model, the accuracy of adding two deep learning algorithms respectively is improved, and the TAL-SRX method, which introduces the two together, has the highest accuracy, which indicates that the Stacking, ANN-LSTM, and Transformer algorithms have their own strengths and positive effects on the specific types of data on the dataset, and that the three algorithms scoring the right samples do not completely cover each other, thus effectively improving the model scoring performance.

### 3.5 Comparative analysis of TAL-SRX methodology and expert scoring prediction results

**Figure 8:**
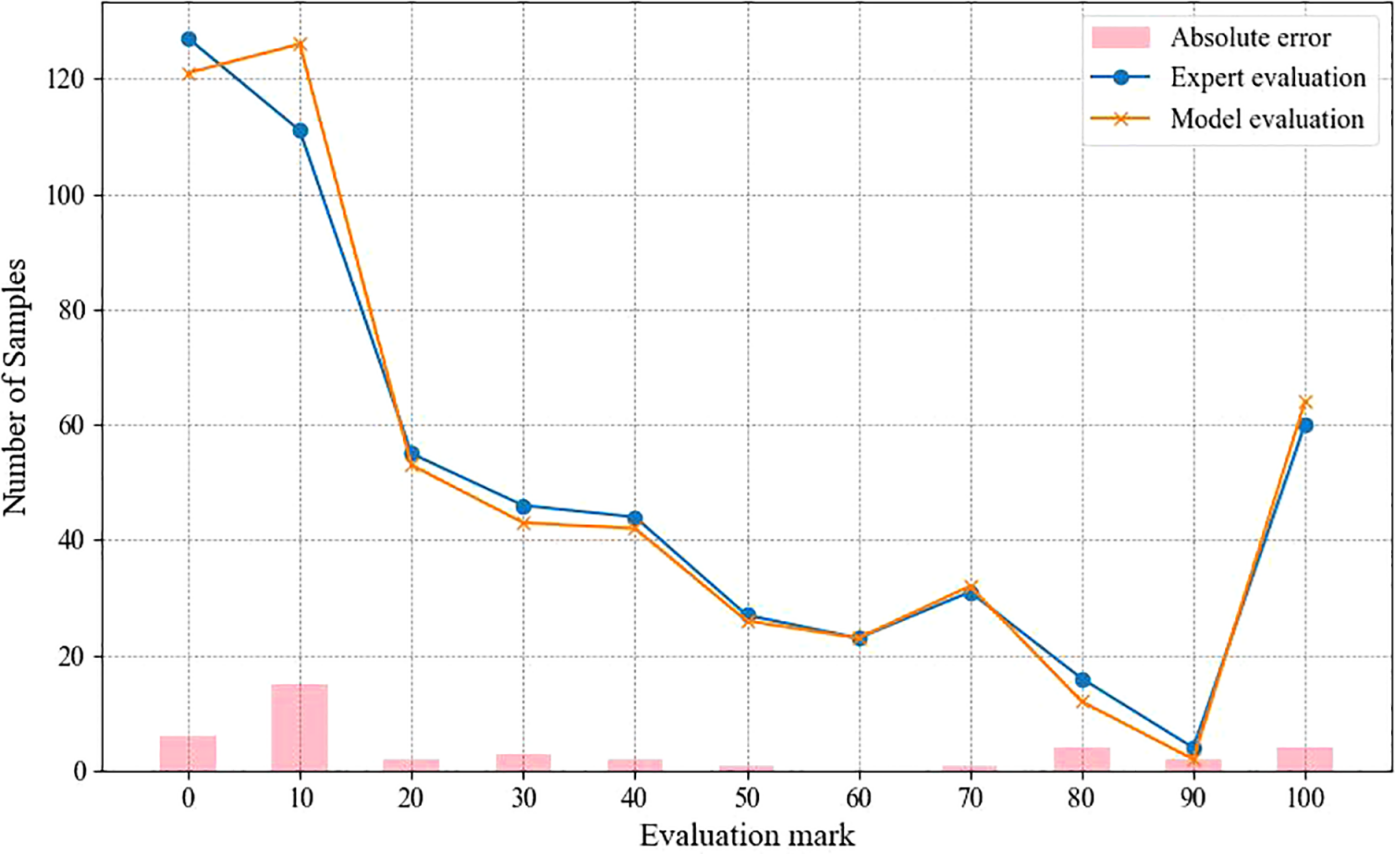
Comparison of TAL-SRX methodology and expert scoring results.

**Figure 8** demonstrates the scoring and sample size relationship curves of expert scoring and TAL-SRX method, which shows that the trajectories of the orange and blue curves are basically the same, and the sample size distribution curves of expert evaluation and algorithmic evaluation have similar trends in each score band, indicating that there is a high degree of consistency between the TAL-SRX evaluation and the expert evaluation, and that the evaluation results of the multi-model fusion method can reflect well the success rate of the development of KASP markers, and then effectively screen out the well-typed markers. However, it is worth noting that there are obvious deviations in the curve trajectories of the two score regions of 0 and 10, which is due to the fact that the Transformer, which has the strongest global feature capturing ability, has the smallest weight in the voting and contributes less to the final prediction results, which makes the performance of the integrated model performance on the samples with dispersed distribution of allele genotypes weaker.

## 4 Discussion

The demand for molecular marker-assisted breeding has driven the development of KASP markers, but the complexity and diversity of allele genotype distribution patterns in the typing results have seriously hindered the large-scale screening of markers, and the existing manual discrimination and algorithms do not have the ability to analyze the data of the typing results with high accuracy and efficiency, making it challenging to develop good molecular markers in bulk. Deep learning, as an important branch of machine learning, has been widely used in various fields, however, there has not been any study using deep learning algorithms to evaluate population genotype amplification and segregation, so it is of great significance to use an integrated strategy to introduce a deep learning model to evaluate the effect of KASP primer typing. In this study, two integration strategies, stacking and soft voting, were used for algorithm combination to construct a multi-model hybrid learning method driven by statistics of KASP marker typing report, and the following conclusions were drawn from the experimental study:

1. Refined the evaluation criteria of KASP primer typing effect, and constructed a competitive allele distribution pattern evaluation system from 0-100 points. This evaluation criterion provides a powerful quantitative tool for analyzing the amplification efficiency, specificity and combinatorial competitiveness of genotypes, which is not only applicable to the results of the KASP test in cotton, but also provides a reference to the resources of other varieties, and can be used to screen for the superior KASP markers that meet the characteristics of different crops.
2. In the multi-model fusion method proposed in this paper, the Stacking integrated learning model is connected using the “base model-metamodel” approach, which gives full play to the respective advantages of heterogeneous learners, and different optimization algorithms are adopted for different learners to accelerate the parameter search process and maximize the performance of each model, and the training process adopts five-fold cross-validation to enhance the model stability. The ANN-LSTM hybrid neural network model combines the ability of nonlinear relationship capture and complex feature extraction, which enhances the adaptability of the model to heterogeneous samples, and the Transformer model is based on the self-attention mechanism, which captures the global dependencies in the high-dimensional feature space, and improves the robustness of TAL-SRX.
3. The proposed model was trained and tested using 3399 sets of KASP marker typing report statistics of cotton varietal resource materials, and the performance comparison between the TAL-SRX method and the base model was carried out firstly, and the TAL-SRX algorithm had a lower error rate and error value than that of the base model, with an accuracy of 92.83% and an AUC value of 0.9905, which was of high evaluative accuracy, consistency and stability, and the performance is significantly better than the single model. Secondly, the impact of each algorithm on the model prediction performance was investigated by integrating and combining ways of ablation comparison, and the experimental results show that the two integration strategies can enhance the model performance, and different sub-algorithms have positive effects on the model. Finally, a comparison between the TAL-SRX method and expert scoring was carried out, and the evaluation results of this method have a high consistency between the number of samples on each score band and the expert evaluation. Therefore, the TAL-SRX method has good evaluation performance.
4. In this study, we explored an intelligent KASP primer typing effect evaluation method using deep learning algorithms and stacking integration, and verified its effectiveness through experiments, which provided the possibility of applying the algorithm to KASP typing data of various crop variety resources.

## 5 Conclusion

In this study, an intelligent typing evaluation method for KASP primers with higher accuracy, called TAL-SRX, is proposed, which can be used to provide more accurate data support for improving the success rate of KASP marker development. The method first introduces an optimization algorithm to construct the Stacking integrated learning framework through three improved models, RS-RF, PSO-SVM and BO-XGBoost, which improves the model prediction performance to some extent and enhances the stability and prediction accuracy of the model. Then, a hybrid neural network is constructed using ANN and LSTM to capture nonlinear relationships and extract complex features to strengthen the model’s ability to adapt to discrepant samples, and the Transformer algorithm with a multi-attention mechanism is introduced to capture the global dependencies in the high-dimensional feature space, which effectively enhances the model’s robustness. Comparison tests were conducted on the test set, and the distribution of the number of samples on each score band was in high agreement between the TAL-SRX method and the expert evaluation results, with an evaluation accuracy of 92.83%, an AUC value of 0.9905, and a lower error rate and error value than that of the base model, and the overall performance was significantly better than that of each single model. In the integrated combination approach ablation comparison test, the TAL-SRX integrated method showed better accuracy and was suitable for KASP primer intelligent typing evaluation. However, it is worth noting that the model proposed in this paper mainly addresses the problem of data evaluation for medium-sized experiments, and is only applicable to the experimental dataset where the mother plate is a 96-well plate, and in the actual operation of KASP, the researchers will also obtain the experimental data of different well plates on a larger scale and a large scale, so it is necessary to further adjust the structure of the model in the subsequent experiments to enhance the generalization ability of the model for more datasets.

## Data Availability

The original contributions presented in the study are included in the article/supplementary material, further inquiries can be directed to the corresponding author/s.
